# Microwave-Assisted Extraction of Cellulose from Aloe Vera Plant Residue and Preparation of Cellulose Nanocrystal–Poly(vinyl alcohol) Hydrogels

**DOI:** 10.3390/molecules29246012

**Published:** 2024-12-20

**Authors:** Eleni Triantafyllou, Andreas Karydis-Messinis, Dimitrios Moschovas, Christina Kyriakaki, Konstantinos C. Vasilopoulos, Aris E. Giannakas, Michael A. Karakassides, Apostolos Avgeropoulos, Nikolaos E. Zafeiropoulos, Constantinos E. Salmas

**Affiliations:** 1 Department of Material Science and Engineering, University of Ioannina, 45110 Ioannina, Greece; triantafyllou.eleni@uoi.gr (E.T.); karydis.and@gmail.com (A.K.-M.); dmoschov@uoi.gr (D.M.); pstm100417@uoi.gr (C.K.); kovasil@auth.gr (K.C.V.); mkarakas@uoi.gr (M.A.K.); aavger@uoi.gr (A.A.); 2 Department of Food Science and Technology, University of Patras, 30100 Agrinio, Greece; agiannakas@upatras.gr

**Keywords:** cellulose, microwave-assisted extraction, cellulose nanocrystals, sulfuric acid hydrolysis, renewable biomass, poly(vinyl alcohol) (PVA), borax, hydrogel

## Abstract

Biomass valorization and bio-based material development are of major research interest following the spirit of the circular economy. Aloe vera cultivation is a widespread agricultural activity oriented toward supplement production because of its well-known antioxidant and antimicrobial properties. Aloe vera juice production also produces a large amount of biomass byproducts that are usually landfilled. On the other hand, cellulose nanocrystals are widely used in several applications, such as biomaterials, bio-compatible polymers, nanocomposites, food packaging, medicines, cosmetics, and sensors, due to their unique physical, mechanical, optical, electrical, and healing properties as well as their compatibility with biological tissues. This study introduces a novel approach combining the microwave-assisted extraction (MAE) of cellulose from this residue with the subsequent isolation of cellulose nanocrystals (CNCs). The MAE process, which exhibits a rapid heating and penetrating ability, was optimized to maximize the cellulose yield under various conditions (microwave power, solvent ratio, and time). Analysis using FTIR, XRD, SEM, and DMA indicated that isolated pure cellulose nanocrystals and a stable PVA–CNC porous hydrogel network were produced. The PVA–CNC hydrogel was synthesized to enable the formation of a semi-crystalline network that imparts the material with enhanced mechanical properties. Both final products of this study could potentially be used for various applications.

## 1. Introduction

Recently, studies on the use of new, non-traditional renewable sources to produce biodegradable nanomaterials have increased. This fact highlights the global effort to reduce the use of conventional sources like wood and cotton. Most natural resources are rich in cellulose, which could potentially be fully utilized to produce cellulose derivatives like cellulose microfibers (CMFs), cellulose nanofibrils (CNFs), and cellulose nanocrystals (CNCs) [1]. While much of the existing research has centered on traditional plant-based resources, this study is oriented to the exploration of underutilized non-conventional sources, such as aloe vera plant residues. Nanocellulose can be derived from various sources like plants, microorganisms, and aquatic animals such as tunicates, all of which are rich in cellulose. Banana leaves, corn cobs, cotton, ramie, rice husks, wood, sugarcane bagasse, sisal leaves, wheat straw, aloe vera leaves, and coconut husks are all promising candidates for the extraction of nanocellulose from plant sources [2,3].

Nanocellulose is a biopolymer consisting of a crystalline fiber structure embedded in an amorphous matrix of lignin, pectin, and hemicellulose. It can be separated primarily into two groups: cellulose nanocrystals (CNCs) and cellulose nanofibers (CNFs) [4]. The categorization of nanocellulose is based on various factors, including nanocellulose’s inherent properties, dimensions, extraction techniques, and intended applications. The properties of these nanomaterials are significantly influenced by the source of cellulose and the methods employed during processing. Although the chemical properties of the cellulose nanocrystals (CNCs), formed via hydrolysis using acid, and the cellulose nanofibers (CNFs), derived via mechanical methods, exhibit similarities, their physical characteristics are different [5]. Several techniques, such as mechanical and/or chemical procedures, have been investigated throughout the years in order to extract CNFs from lignocellulosic biomass [6]. CNCs, also known as cellulose substances, are categorized as sustainable bio-based nanomaterials generated from the degradation of the lignocellulosic biomass. Nanoscale dimensions with numerous intrinsic properties make them suitable for various applications, such as packaging and biomedical applications [7]. Additionally, their inherent properties, including low density, renewability, biodegradability, adjustable surface chemistry, non-toxicity, and increased thermal stability, are significant. Each of these properties plays a crucial role in various fields, particularly in the context of material science, environmental sustainability, and health. By processing these properties, materials can be engineered to meet specific performance requirements while minimizing negative impacts on human health and the environment [7].

The MAE setup and parameters are provided in Section 3.2. The cellulose, CNC samples, and CNC–PVA hydrogel were thoroughly analyzed using Fourier-transform infrared spectroscopy (FTIR), X-ray diffraction (XRD), scanning electron microscopy (SEM), and dynamic mechanical analysis (DMA) [8].

Hydrogels are polymeric materials that have a 3D structure and the ability to absorb and reversibly release large amounts of water and biological fluids [9]. The hydrogels can also respond to various condition changes, such as pH, temperature, ions, and magnetic or electric fields [10]. According to the origin of the polymers, hydrogels can be divided into natural and synthetic hydrogels. Those based on natural polymers derive from hydrophilic polymers, such as cellulose, gelatine, chitosan, hyaluronic acid, and some of their derivatives. In addition to natural polymers, there are also hydrogels based on synthetic polymers, which typically include polymers such as poly(ethylene glycol) (PEG), poly(acrylic acid) (PAA), polyacrylamide (PAAm), poly(vinyl alcohol) (PVA), and their copolymers [11].

PVA, a synthetic polymer, is both safe and eco-friendly as it is designed to degrade naturally. Its versatility has led to its adoption across diverse industries, such as medicine, packaging, food production, and papermaking [12]. Moreover, there is the potential for PVA to be applied in biomedical uses like tissue engineering, cell cultures, and crafting implants for vascular systems. It has been combined with a range of fibers and micro-sized cellulose-based materials, such as cellulose nanocrystals (CNCs), to improve its properties. Given that CNCs are hydrophilic, PVA–CNC hydrogels are an environmentally friendly option for biomaterial development. These hydrogels can be customized to be lightweight and biodegradable [13]. PVA and CNC interact via secondary interactions, which enhances the mechanical properties of the hydrogel [14].

Cellulose-based hydrogels present characteristics that make them suitable for use in biomedical applications. Cellulose is non-toxic, which is essential for any material intended for use in biomedical applications. Cellulose-based hydrogels typically exhibit a high water absorption capacity due to the hydrophilic nature of cellulose [15], which enables them to retain a significant amount of water, making them suitable for applications such as wound dressings, where moisture management is crucial for healing promotion. They can also be engineered to have a wide range of mechanical properties, porosities, and degradation rates, depending on the application. Overall, their properties, such as biodegradability, biocompatibility, non-toxicity, hydrophilicity, and structural versatility, make them possible candidates for numerous biomedical applications, offering potential benefits in terms of safety, efficacy, and performance [16]. When incorporated into hydrogels, cellulose acts as a reinforcing material, enhancing the mechanical strength of the hydrogel matrix. The presence of cellulose fibers or nanoparticles within the hydrogel network helps to distribute mechanical stresses more effectively, resulting in improved elasticity and structural integrity. Furthermore, cellulose’s ability to form hydrogen bonds with water molecules contributes to the hydrogel’s stability and swelling behavior. The hydrophilic nature of cellulose facilitates water absorption, which is beneficial for applications such as tissue engineering and drug delivery [17].

By incorporating therapeutic agents, such as growth factors or anti-inflammatory drugs, into the hydrogel matrix, controlled release can be achieved, aiding in tissue regeneration and reducing inflammation in the affected area. Moreover, hydrogels can be combined with other biomaterials, such as ceramics or polymers, to create composite materials with enhanced properties suitable for specific bone repair applications. These composite materials can offer a synergistic approach to addressing complex bone defects or injuries [18].

An additional feature is that “smart” cellulose hydrogels can be created that are capable of being used as drug carriers, where proteins and peptides will be protected from the host environment but will be capable of allowing for the acceleration as well as the retardation of their release, as the polymer chains will expand or contract [19]. “Smart” cellulose hydrogels can be engineered to function as advanced drug carriers with controlled release capabilities. These hydrogels possess the ability to respond to various stimuli, such as pH, temperature, or specific biochemical signals, allowing for precise modulation of drug release kinetics. The expansion and contraction of polymer chains within the hydrogel matrix plays a crucial role in achieving controlled release profiles [20]. This results in the ability to remotely control drug delivery from a control switch, thus presenting a significant advantage in many biomedical applications. Finally, cellulose nanocrystals, which are isolated through the acid hydrolysis of native cellulose, can be used to reinforce the polymeric hydrogel due to its mechanical properties [21].

In this study, biomass derived from the valorization of the well-known aloe vera plant was further processed to produce CNCs via a novel microwave-assisted extraction (MAE) technique. Microwave-assisted extraction (MAE) processes were recently developed and demonstrate advantages that overcome possible issues with the traditional approaches. These advantages include enhanced reproducibility and minimal sample manipulation, a reduced solvent volume, a decreased exposure time, and lower energy consumption during the extraction process [22]. The aim of the present study was the development of a green technique for the extraction of CNCs from aloe vera leaves via a cost-effective MAE process and overcoming the major drawbacks of traditional extraction procedures [23]. A second target of this study was the preparation of a semi-crystalline PVA–CNC hydrogel network. Such materials are already known to scientific audiences and some publications refer to a similar development [24,25]. Nevertheless, the novelty of our work is the derivation of CNCs from aloe vera biomass and the use of the MAE technique for the extraction of these crystals.

## 2. Results and Discussion

### 2.1. FT-IR Spectrum

**Figure 1:**
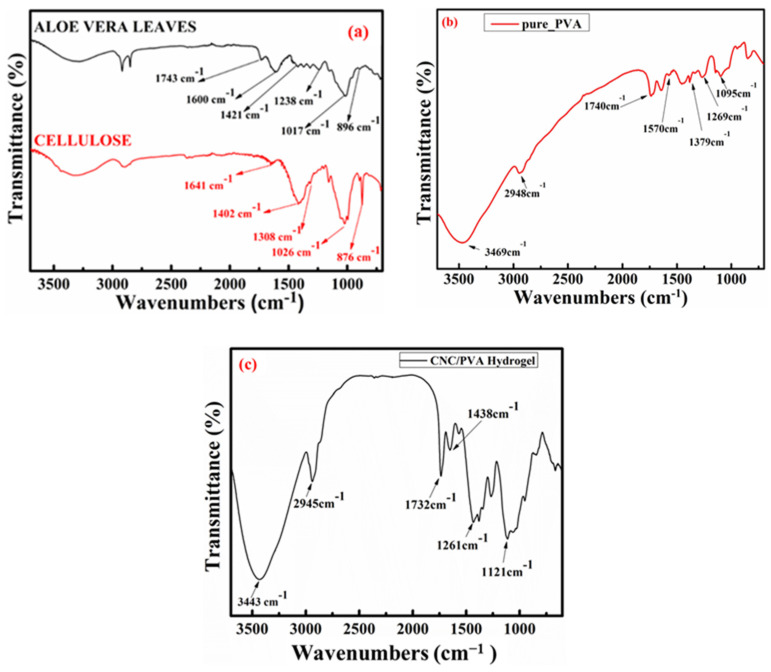
FTIR spectra of (**a**) pure aloe vera leaves and extracted cellulose and (**b**) pure PVA powder and (**c**) CNC–PVA hydrogel.

In Figure 1a, the FTIR spectrum of the aloe vera samples displays several key bands indicating specific functional groups. The ether R-O-R group is identified by a peak at 1017 cm^−1^, while the secondary alcohol R-OH group appears at 1238 cm^−1^. Additionally, an ether R-O-O-R group is noted at 1230 cm^−1^, and an aromatic group shows a band at 1421 cm^−1^. A notable absorption at 1600 cm^−1^ indicates the presence of a nitro (NO_2_) group, alongside a strong C=C stretching band, which suggests the presence of vinyl ether and aloin compounds in the aloe vera gel [25].

Further, characteristic peaks between 3660 and 2900 cm^−1^ correspond to the stretching vibrations of O-H and C-H bonds typically found in polysaccharides. The broad absorption around 3331 cm^−1^ is attributed to the hydroxyl group stretching within polysaccharides, also reflecting both inter- and intra-molecular hydrogen bonding in cellulose. Additional cellulose-specific bands appear in the 1630–900 cm^−1^ range, with a distinct peak at 1641 cm^−1^, likely representing water molecules absorbed by the cellulose structure. Other notable bands include those at 1402, 1308, 1026, and 876 cm^−1^, which correspond to stretching and bending vibrations in -CH_2_, -CH, -OH, and C-O bonds within the cellulose matrix [26]. Figure 1b illustrates the FTIR spectrum for pure PVA powder. For PVA, the characteristic absorption peaks are at 3469 cm^−1^ (O-H stretching), 2948 cm^−1^ (asymmetric stretching of CH_2_), 1740 cm^−1^ (due to water absorption), 1570 cm^−1^ (CH_2_ bending), 1379 cm^−1^ (OH rocking with CH wagging), 1269 cm^−1^ (shoulder stretching of C-O) (the crystalline sequence of PVA), and 1095 cm^−1^ (stretching of C=O and bending of OH) (the amorphous sequence of PVA) [26].

In Figure 1c, the FTIR spectrum of the PVA–CNC hydrogel reveals a prominent band at approximately 3443 cm^−1^ in all spectra, indicating the free O-H stretching vibration of hydroxyl (OH) groups. This band is primarily due to intra-molecular hydrogen bonding within the PVA, as well as inter-molecular hydrogen bonding between the hydroxyl groups of PVA and CNCs, with the addition of CNCs resulting in a shift toward a higher wavenumber. The C-H stretching vibrations from alkyl groups are detected at 2945 cm^−1^ across all hydrogel formulations. The peak at 1732 cm^−1^ is associated with C=O stretching, representing residual acetate groups in the PVA matrix; however, its intensity decreases upon the addition of CNCs, likely due to the new hydrogen bonds formed between PVA and CNC molecules. The band observed at 1261 cm^−1^ corresponds to C-O stretching vibrations, with the reduced intensity again reflecting hydrogen bonding interactions between PVA and CNCs. The peak at 1438 cm^−1^ is attributed to C-H deformation vibrations, while the peak at 1121 cm^−1^ likely results from C-H bending in the CH_2_ groups. These observations suggest a robust interaction between PVA and CNCs within the hydrogel structure, contributing to the hydrogel’s stability and mechanical properties [27].

### 2.2. XRD Analysis

X-ray diffraction (XRD) analysis of aloe vera leaves typically reveals diffraction peaks corresponding to the crystalline structure of the materials present in the leaves. Aloe vera leaves are composed of various compounds, including cellulose, hemicellulose, lignin, and other polysaccharides.

**Figure 2:**
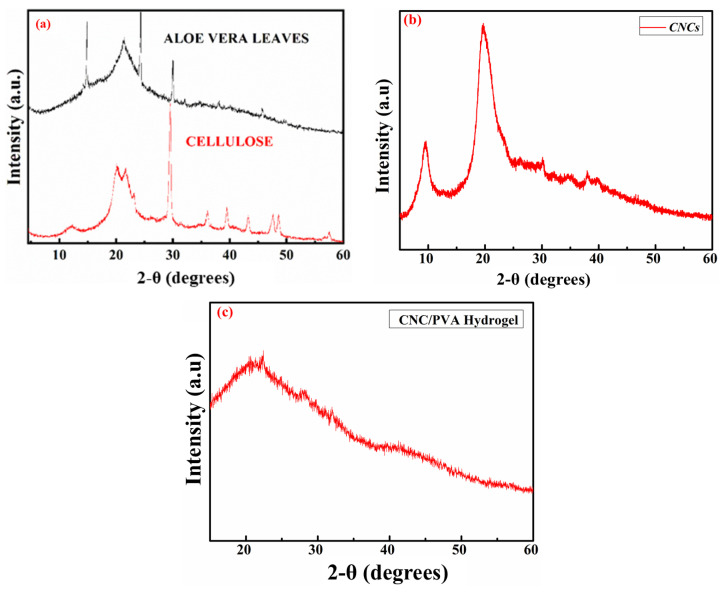
XRD diffractograms of (**a**) pure aloe vera leaves and extracted cellulose, (**b**) CNCs, and (**c**) the CNC–PVA hydrogel.

The XRD pattern may show peaks associated with the crystalline components, such as cellulose, which usually appear around 2θ values of 14.8°, 16.5°, and 23.6°. These peaks can provide information about the crystallinity and structural properties of the cellulose and other crystalline components in the aloe vera leaves [27]. X-ray diffraction (XRD) analysis of cellulose typically shows diffraction peaks indicative of its crystalline structure. The XRD pattern usually displays sharp peaks at specific 2θ values, typically around 18.8°, 22°, and 29.7°. The intensity and position of these peaks can provide information about the crystallinity, crystal size, and orientation of the cellulose molecules within the sample [28]. The PVA–CNC hydrogel exhibits a diffraction peak at 2θ = 21° with decreased density and a shoulder peak at 2θ = 28.2° with increased density, which suggests the physical interaction of PVA and CNCs. Figure 2b shows an XRD graph of the CNCs that exhibits a prominent diffraction peak around 22°, which is characteristic of the crystalline structure of cellulose and corresponds to the crystallographic plane in cellulose. A smaller, broader peak around 15° indicates the presence of the amorphous phase, suggesting a mix of crystalline and amorphous regions in the material. The gradual decline in intensity at higher angles (above 30°) further supports the predominantly crystalline nature with minor amorphous contributions. This pattern confirms the semi-crystalline structure typical of CNCs, with a high degree of crystallinity and distinct ordering [29].

Figure 2c indicates that the incorporation of CNCs and PVA does not affect the crystalline structure of the PVA matrix. This means that the PVA was well dispersed in the CNC suspension in order to form the PVA–CNC hydrogel [30].

The significant peaks observed around 2θ angles, typically near 22.5° and 16°, reflect the crystalline regions of cellulose, which is the dominant form in CNCs. These peaks indicate the degree of crystallinity in the CNCs, which is closely related to their mechanical strength, thermal stability, and rigidity. The crystallinity index, derived from the intensity of these peaks, provides insight into the quality and purity of CNCs. Enhanced crystallinity is directly linked to the performance of CNCs in composite materials, as it contributes to the stiffness, the transparency, and improved reinforcement properties when incorporated into matrices like PVA.

### 2.3. Scanning Electron Microscopy (SEM)

**Figure 3:**
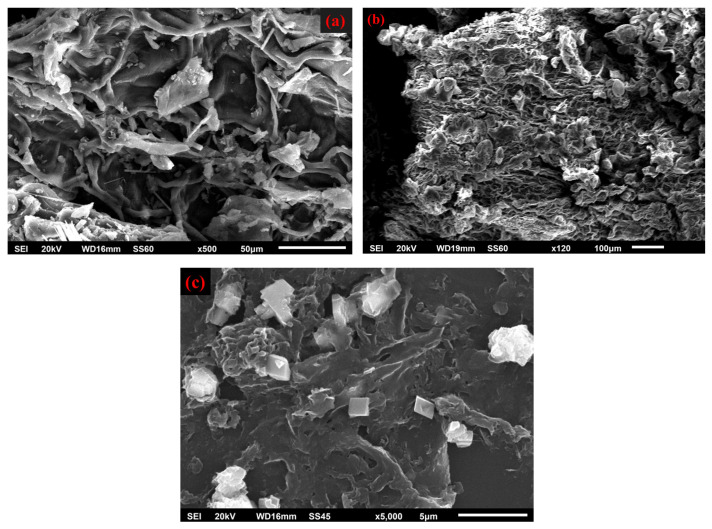
SEM images of (**a**) pure aloe vera leaves, (**b**) extracted cellulose, and (**c**) isolated CNCs.

Figure 3a illustrates the amorphous structure and surface morphology observed through SEM analysis. The micrograph highlights surface holes indicative of the material’s inherent porosity. These images reveal a porous and irregular surface characterized by heterogeneous structural features. Bleaching with H_2_O_2_ after the hydrothermal treatment helps to eliminate the rest of the lignin and with further disintegration leading to the development of cellulose microfibrils. Figure 3b displays a fibrous and layered structure. This could suggest the presence of cellulose microfibrils. The bleached pulp exhibits a smoother and uniform fibril surface, confirming the removal of non-cellulosic components. Morphological examination of the CNCs is essential because the source of cellulose and the hydrolysis technique have a profound influence on the dimensions and properties of nanocellulose.

The morphology observed in the SEM image (Figure 3c) consists of irregularly shaped features with varying dimensions. Some features show an aggregated or clustered appearance, which is consistent with the air-drying process leading to the compaction of nanoscale particles. The contours of the features suggest a rough surface texture, possibly indicating closely packed crystalline material. The aggregation observed masks individual nanocrystals. Overall, the morphology points to a sample with a heterogeneous distribution of shapes and sizes, potentially influenced by the preparation method.

**Figure 4:**
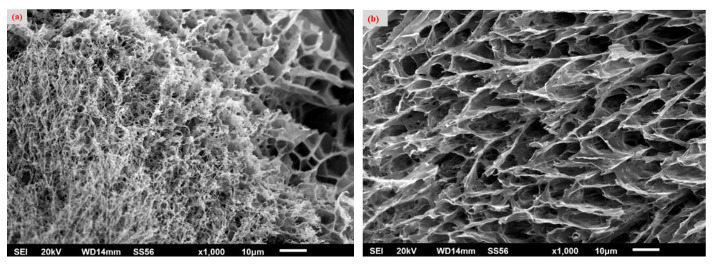
SEM images of the CNC–PVA hydrogel, (**a**) sponge morphology and (**b**) interconnected porous system.

To study the interior structure of the hydrogel, it was freeze-dried to prevent shrinking and examined using SEM imaging. Figure 4 shows that the hydrogel produced an open, interconnected, continuous, and macroporous structure. Figure 4a shows tiny extensions reaching outwards from the matrix cell walls. It is possible that these projections are CNC whiskers. There are no such projections visible in Figure 4b; instead, lengthy filaments extending across many cells can be detected on occasion [31].

### 2.4. Dynamic Mechanical Analysis (DMA)

**Figure 5:**
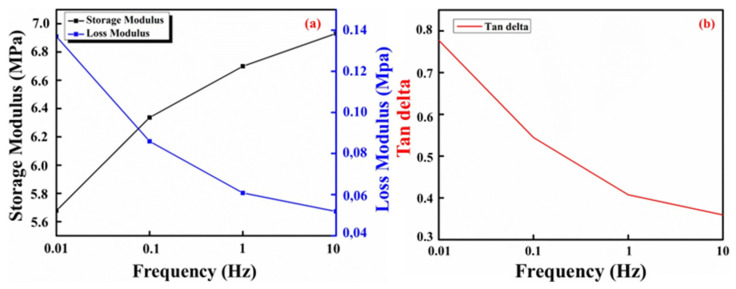
Dynamic mechanical analysis of the CNC–PVA hydrogel, (**a**) Storage and Loss Modulus and (**b**) Tan delta are plotted as function of Frequency.

Frequency sweeps were performed on the CNC–PVA hydrogel. The storage modulus and loss modulus obtained from dynamic frequency sweep measurements can give further information about the microstructure of a CNC–PVA hydrogel, as shown in Figure 5a. It shows that, for a fixed temperature (35 °C), the storage modulus rises as the frequency increases. The increase in the storage modulus as a function of frequency indicates that the hydrogel retains a strong network structure while becoming more elastic and producing stable structures. Increasing the crosslinking density enhances the network’s mechanical strength, leading to a higher storage modulus. Additionally, high crosslinking density restricts chain mobility, resulting in reduced energy dissipation and thus a lower loss modulus. The restriction of the movement of polymer chains and the reduction of their ability to dissipate energy result in a decrease in the tan delta as shown in Figure 5b [32].

## 3. Materials and Methods

### 3.1. Materials

Sigma–Aldrich (St. Louis, MO, USA) was the supplier of Sodium Hydroxide (NaOH, 96%), Hydrogen Peroxide (H_2_O_2_, 30%), poly(vinyl alcohol) (PVA 87–90% Hydrolyzed), Sodium Tetraborate Decahydrate (borax decahydrate), and Sulfuric Acid (H_2_SO_4_, 98%). Aloe vera (AV) waste leaves were supplied by the Greek Industrial Company, Hellenic Aloe, Ethnikis Antistaseos 21, Heraklion, Crete, Greece.

### 3.2. Cellulose Extraction

**Figure 6:**
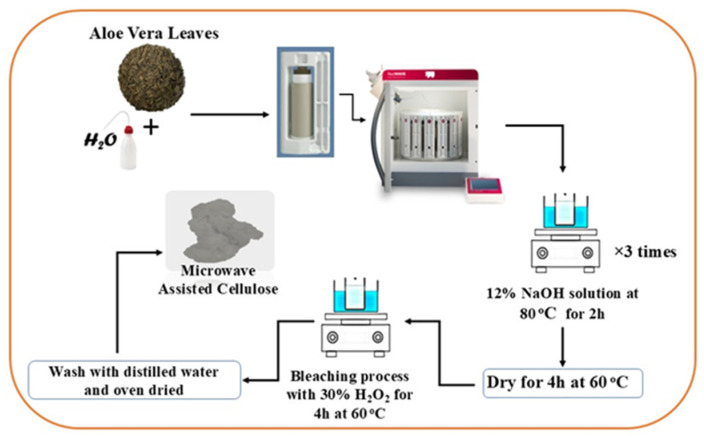
Schematic illustration of cellulose extraction.

AV waste leaves were cut into small pieces and dried in an oven at 60 °C for 4 h under vacuum. Then, the dried AV leaves were blended in a blender until a powder was collected. The collected powder (2 g) and 50 mL of distilled water were immersed in a 100 mL Teflon vessel. The vessel was placed in the microwave at 200 °C for 15 min. A pressure of 25 bar was reached at 1080 Watts. After the microwave treatment, the sample was filtered with filter paper. Then, a beaker containing 50 mL of 12% NaOH solution was heated at 80 °C for 2 h. This procedure was repeated three times, the mixture was filtered, and the solid sample was dried at 60 °C for 4 h. The obtained sample was bleached with 30% H_2_O_2_ for 4 h at 60 °C. Then, the bleached product was washed multiple times with distilled water and placed in an oven to dry (Figure 6).

### 3.3. Cellulose Nanocrystal Isolation

**Figure 7:**
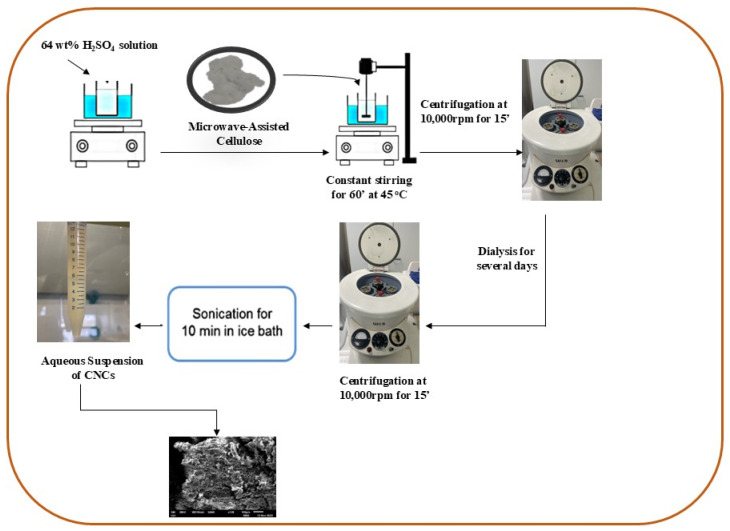
Schematic illustration of cellulose nanocrystal isolation.

The extraction of cellulose nanocrystals (CNCs) was achieved by subjecting the isolated cellulose microfibrils (CMFs) to sulfuric acid hydrolysis. In this process, CMFs were carefully introduced into a preheated sulfuric acid solution (64 wt%) maintained at 50 °C, with continuous mechanical stirring to ensure uniform exposure and a uniform reaction. After a designated period, the reaction was promptly halted by diluting the mixture with cold distilled water, which effectively quenched the acid hydrolysis. The resulting suspension was then centrifuged at 12,000 rpm for 15 min, which was repeated several times to separate the solid CNCs from the liquid phase. Following centrifugation, the mixture underwent dialysis against distilled water until a neutral pH was reached, removing the excess acid and other impurities. Finally, to ensure a uniform CNC dispersion, the aqueous CNC suspension was homogenized using a probe-type ultrasonic homogenizer for 5 min, as illustrated in Figure 7. This ultrasonic treatment further stabilized the CNCs in the suspension, preparing them for subsequent applications or analysis [29].

### 3.4. PVA–CNC Hydrogel Preparation

**Figure 8:**
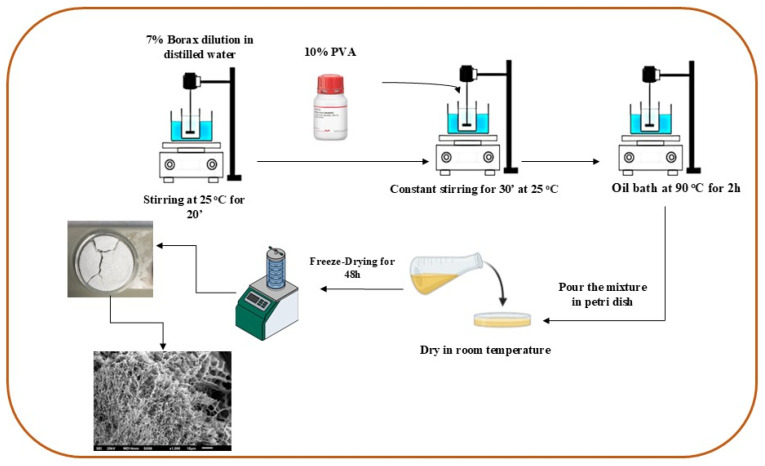
Schematic illustration of CNC–PVA hydrogel preparation.

To prepare the PVA–CNC hydrogel, a borax solution (7 wt%) was first diluted into a beaker containing the CNC suspension (50 mL, 0.5 wt% CNCs), with the mixture stirred continuously at 25 °C for 20 min to ensure an even distribution of the borax. Following this, poly(vinyl alcohol) (PVA) solution (10 wt%) was gradually added to the same beaker in a proportion that maintained a CNC-to-PVA ratio of 5% (*w*/*w*). The mixture was stirred for an additional 30 min, allowing the PVA to fully dissolve and integrate into the suspension. Once the PVA was completely dissolved, the beaker was transferred to an oil bath, where the mixture was heated to 90 °C and stirred continuously for 2 h. This heating step facilitated crosslinking between the components, contributing to the formation of the hydrogel structure. Afterward, the mixture was poured into a petri dish and allowed to dry at room temperature, enabling initial gel setting. To preserve the hydrogel and achieve a porous structure, the sample was stored at −20 °C and then subjected to lyophilization, resulting in a stable, porous hydrogel network. This process is illustrated in Figure 8.

## 4. Conclusions

The hydrogels synthesized in this study from aloe vera biomass waste exhibit promising characteristics due to their biopolymer-based composition. These hydrogels, which were formed using the solution casting and evaporation method with PVA and borax as a crosslinker, showed favorable mechanical and swelling properties that make them suitable for various applications. The biodegradability of the final product, the natural origin of most of the precursor materials, the valorization of plant biomass that would otherwise become landfill, the well-known antioxidant and antimicrobial properties of the aloe vera plant, and the presence of cellulose nanocrystals contribute to the economic and physical viability of the process. The final material produced from such a process is a novel material with enhanced strength, stability, and antimicrobial and antioxidant characteristics and is a potential candidate for the applications mentioned in the Introduction section. In the future, our team will carry out research activities on the ability of this material to be loaded with bioactive compounds and on its potential for controlled release in drug delivery systems. Further antioxidant, antimicrobial, and food preservation tests will confirm its suitability for active food packaging and food shelf-life extension. This ongoing research will further demonstrate its viability as an eco-friendly alternative solution for issues in these fields.

## Data Availability

Data is contained within the article.
